# The Prevalence of Low Back Pain Among Medical Students: A Cross-Sectional Study From Saudi Arabia

**DOI:** 10.7759/cureus.38997

**Published:** 2023-05-14

**Authors:** Youssef A Taha, Hadi A Al Swaidan, Hadi S Alyami, Muhannad M Alwadany, Mohammad H Al-Swaidan, Yahya H Alabbas, Hassan M Dhaen, Abdullah A Faidhi

**Affiliations:** 1 General Practice, King Khalid Hospital, Najran, SAU; 2 General Practice, King Faisal University, Hofuf, SAU; 3 General Practice, Arabian Gulf University, Manama, BHR

**Keywords:** saudi arabia, cross-sectional survey, risk factors, prevalence, medical students, low back pain

## Abstract

Background: Low back pain is a prevalent and debilitating condition that affects a significant proportion of the adult population. Medical students are particularly vulnerable due to the demands of their rigorous curriculum. Therefore, this study aims to investigate the prevalence and risk factors associated with low back pain among medical students.

Methods: A cross-sectional survey was conducted among medical students and interns at King Faisal University in Saudi Arabia using a convenience sampling technique. An online questionnaire was distributed via social media applications to explore the prevalence and risk factors for low back pain.

Results: Out of 300 medical students who participated in the study, 94% reported experiencing low back pain, with a mean pain score of 3.91 ± 2.0 out of 10. The most common factor that aggravated the pain was prolonged sitting. Logistic regression analysis revealed that sitting for more than eight hours (OR=5.61; 95% CI: 2.92-21.42) and not engaging in physical exercise (OR=3.10; 95% CI: 1.34-6.57) were independently associated with a higher prevalence of low back pain. These findings highlight the increased risk of low back pain among medical students due to prolonged sitting and a lack of physical activity.

Conclusion: This study provides evidence of the high prevalence of low back pain among medical students and identifies significant risk factors that exacerbate the condition. It emphasizes the need for targeted interventions to promote physical activity, reduce prolonged sitting, manage stress, and encourage good posture among medical students. The implementation of such interventions could help alleviate the burden of low back pain and improve the quality of life for medical students.

## Introduction

Low back pain is a widespread health problem that affects people of all ages and professions. It is estimated that up to 80% of adults will experience low back pain at some point in their lives, making it one of the most common musculoskeletal disorders worldwide [1]. The economic burden of low back pain is substantial, with direct and indirect costs estimated to be billions of dollars annually [1]. Moreover, low back pain is a leading cause of disability globally, with profound impacts on quality of life, productivity, and mental health [1,2].

Medical students, in particular, are at high risk for developing low back pain due to the demanding nature of their curriculum [2]. Medical students spend extended hours studying, attending lectures, and performing clinical rotations, which can result in prolonged sitting or standing postures, repetitive motions, and awkward body positions. Previous studies have reported a high prevalence of low back pain among medical students, ranging from 30% to 75%, depending on the study population and diagnostic criteria [2-5]. The incidence of low back pain among medical students is also reported to increase with academic progression, with the highest rates reported in the later years of medical school.

The consequences of low back pain among medical students can be substantial, as it can have negative impacts on their academic performance, career prospects, and mental health [6,7]. Medical students with low back pain may experience reduced concentration, poor sleep quality, and limited physical activity, which can lead to decreased academic performance and increased stress levels. Moreover, low back pain may cause medical students to miss classes, rotations, and exams, further compromising their academic and professional goals [2-5].

Given the potential impact of low back pain on medical students, there is a need to identify risk factors and effective interventions for preventing and managing the condition in this population. Understanding the factors associated with low back pain among medical students can aid in the development of targeted interventions that address modifiable risk factors such as poor posture and sedentary behavior. Additionally, identifying effective interventions for managing low back pain can improve the quality of life and academic performance of medical students while reducing the burden of low back pain on healthcare resources. Therefore, this study aims to investigate the prevalence, risk factors, and management strategies for low back pain among medical students.

## Materials and methods

Study design

This cross-sectional survey was conducted among medical students and interns at King Faisal University in Saudi Arabia between September 2022 and October 2022.

Study population

Participants included medical students and interns from the second to sixth year at King Faisal University, aged 18 years and above. Exclusion criteria were medical students or interns outside of King Faisal University, those with trauma-related pain, and those with incomplete responses. Participants were selected using a convenience sampling technique, whereby available and willing participants were included in the study.

Sample size calculation

The sample size was calculated using the following parameters: a population of 950, a 95% confidence level, a response distribution of 50%, and a margin of error of no more than 5%. Based on this, the estimated sample size was 274 participants. To increase the accuracy of the results, we included 300 participants in our study.

Data collection

An online questionnaire was developed using Google Forms and distributed via social media applications such as WhatsApp, Facebook, and Instagram. The questionnaire was available in both English and Arabic. To avoid duplication of responses, each participant was restricted to filling out the questionnaire only once. The survey was composed of three sections: the first part included demographic data such as age, gender, height, weight, body mass index, grade point average (GPA), academic year, and history of low back pain; the second part focused on the characteristics of the pain such as the duration, frequency, severity, and location of the pain; and the third part focused on the risk factors, signs, and symptoms of back pain such as posture, physical activity, smoking, and stress. The 10-point pain score was used to assess the severity of the pain. This score is a simple numerical rating system used to measure the intensity of pain. It involves asking the person experiencing pain to rate the severity of their pain on a scale from 0 (no pain) to 10 (worst possible pain).

Data analysis

The data collected were analyzed using IBM SPSS version 24 (IBM SPSS, Armonk, NY), which is a statistical software package used for analyzing data. A descriptive analysis was conducted to calculate measures such as the mean, median, mode, frequency, and standard deviation. Categorical variables were analyzed using the chi-squared test, which is a statistical method used to test the association between two categorical variables. The significance level (P ≤ 0.05) was considered the cutoff point for all statistical procedures.

Ethical considerations

Ethical clearance for this study was obtained from the ethical committee of the Medicine College at King Faisal University. All participants were informed of the study's objectives, and their participation was voluntary. The questionnaire included a consent form that participants had to read and sign before answering the questions. The data collected were kept confidential, and only the research team had access to them. The participants' privacy and anonymity were maintained throughout the study, and their names and other personal identifiers were not recorded. Any data collected were only used for research purposes and were not shared with any other individual or organization.

## Results

Baseline characteristics of participants

The study included 300 medical students with a mean age of 22.58 ± 2.07 years. Of the total participants, 170 (56.7%) were male, 88 (29.3%) had a body mass index greater than 25, 109 (36.3%) had a GPA of 4-4.49, and 93 (31%) belonged to the fourth academic year (Table 1).

**Table 1:** Baseline characteristics. GPA: grade point average.

Characteristics		Frequency	Percent
Gender	Female	130	43.3
	Male	170	56.7
Mean age	22.58 ± 2.07 years		
Body Mass Index	Underweight	30	10.0
	Normal weight	182	60.7
	Overweight	67	22.3
	Obesity	21	7.0
GPA	<3	17	5.7
	3-3.49	32	10.7
	3.5-3.99	73	24.3
	4-4.49	109	36.3
	4.5-5	69	23.0
Academic year	Second year	41	13.7
	Third year	59	19.7
	Fourth year	93	31.0
	Fifth year	33	11.0
	Sixth year	36	12.0
	Intern	38	12.7

Prevalence and characteristics of lower back pain

Among the participants, 282 (94.0%) reported low back pain, and of those, 254 (90.1%) had low back pain not related to trauma. The mean pain score was 3.91 ± 2.0. Dull pain was reported by 78 (27.7%) participants, and 65 (23%) reported cramping pain. Long sitting was the most commonly reported factor that increased pain (39%), followed by stress (37.6%), wrong sleep position (36.5%), physical inactivity (35.1%), and anxiety (29.8%). The most commonly reported methods to decrease pain were sleeping in the right position (45%), maintaining good posture (44.3%), sitting in the right position (41.5%), physiotherapy (19.5%), applying heat (19.5%), and applying ice (16.7%) (Table 2).

**Table 2:** Prevalence of low back pain and associated features.

Variable		Frequency	Percent
Have low back pain	No	18	6.0
	Yes	282	94.0
History of trauma	No	254	90.1
	Yes	28	9.9
Duration	<3 months	196	69.5
	≥3 months	86	30.5
Factors increase pain	Anxiety	84	29.8
	Stress	106	37.6
	Backpack	76	27.0
	Intensive sport activity	61	21.6
	Wrong sleep position	103	36.5
	Long sitting session	110	39.0
	Inappropriate lifting	54	19.1
	Physical inactivity	99	35.1
Methods used to decrease pain	Physiotherapy	55	19.5
	Applying heat	55	19.5
	Applying ice	47	16.7
	Maintain good posture	125	44.3
	Sit in the right position	117	41.5
	Sleeping in the right position	127	45.0
	Nothing	33	11.7

Of the participants who experienced low back pain without trauma (n=254), the most common condition associated with low back pain was neck pain (37%), followed by low mood (36.6%), difficulty at work (31.5%), and weakness (28.3%). Only 19 (7.5%) used medication for their low back pain. Approximately 187 (73.6%) reported studying in a sitting position, and 91 (35.8%) spent two to four hours sitting. Among those who had low back pain without a history of trauma, only 15 (5.9%) reported lifting heavy objects, 47 (18.5%) smoked cigarettes, 204 (80.3%) drank coffee, and 137 (53.9%) had a family history of low back pain. Low back pain affected daily activities for 139 (54.7%) participants, and 109 (42.9%) reported that it affected their GPA (Table 3).

**Table 3:** Clinical features of non-traumatic low back pain among students. GPA: grade point average.

Variable		Frequency	Percent
Associated symptoms	Numbness	59	23.2
	Weakness	72	28.3
	Neck pain	94	37.0
	Leg weakness	40	15.7
	Low mood	93	36.6
Take medications	No	235	92.5
	Yes	19	7.5
Study position	Lying down	38	15.0
	Sitting	187	73.6
	Walking	29	11.4
Time spent sitting	1-2 hours	46	18.1
	2-4 hours	91	35.8
	4-8 hours	76	29.9
	More than 8 hours	41	16.1
Time spent for exercises	1-3 hours/week	94	37.0
	>3 hours/week	31	12.2
	Never	129	50.8
Lift heavy object	Never	64	25.2
	Rarely	134	52.8
	Regularly	15	5.9
	Usually	41	16.1
Smoking	No	207	81.5
	Yes	47	18.5
Regular coffee intake	No	50	19.7
	Yes	204	80.3
Pain affects daily activities	No	115	45.3
	Yes	139	54.7
Low back pain affected GPA	No	145	57.1
	Yes	109	42.9
Family history of back pain	No	117	46.1
	Yes	137	53.9
Seek medical advice	No	187	73.6
	Yes	67	26.4

Association of low back pain with baseline characteristics

The prevalence of low back pain was significantly lower in underweight students and higher in those who were overweight and obese (p=0.049). No significant association was found between low back pain and the gender, GPA, and academic year of the students (p>0.05) (Table 4).

**Table 4:** Relationship between low back pain and baseline characteristics.

Variable			Low back pain		P-value
			No	Yes	
Gender	Female	N	8	122	0.922
		%	6.2%	93.8%	
	Male	N	10	160	
		%	5.9%	94.1%	
Body Mass Index	Underweight	N	5	25	0.049
		%	16.7%	83.3%	
	Normal weight	N	7	175	
		%	3.8%	96.2%	
	Overweight	N	5	62	
		%	7.5%	92.5%	
	Obesity	N	1	20	
		%	4.8%	95.2%	
Grade point average (GPA)	<3	N	2	15	0.707
		%	11.8%	88.2%	
	3.00–3.49	N	1	31	
		%	3.1%	96.9%	
	3.50–3.99	N	3	70	
		%	4.1%	95.9%	
	4.00–4.49	N	7	102	
		%	6.4%	93.6%	
	4.50–5.00	N	5	64	
		%	7.2%	92.8%	
Academic year	Second year	N	3	38	0.831
		%	7.3%	92.7%	
	Third year	N	4	55	
		%	6.8%	93.2%	
	Fourth year	N	4	89	
		%	4.3%	95.7%	
	Fifth year	N	3	30	
		%	9.1%	90.9%	
	Sixth year	N	1	35	
		%	2.8%	97.2%	
	Intern	N	3	35	
		%	7.9%	92.1%	

Logistic regression analysis was performed to predict the risk factors for low back pain in medical students. Sitting for more than eight hours (OR=5.61; 95% CI: 2.92-21.42) and doing no exercise (OR=3.10; 95% CI: 1.34-6.57) were found to be independently associated with a higher prevalence of low back pain (Table 5).

**Table 5:** Regression model for low back pain risk factor analysis.

Variable	Odds ratio	95% Confidence Interval		P value
		Lower	Upper	
Gender	0.96	0.27	3.33	0.944
BMI >25	0.47	0.14	1.59	0.224
Study in the sitting position	0.77	0.36	1.63	0.491
Sitting >8 hours/day	5.61	2.92	21.42	0.029
Lack of exercise	3.10	1.34	6.57	0.016
Lift heavy objects regularly	0.93	0.23	3.83	0.922
Smoking	1.25	0.24	6.63	0.794
Coffee intake >4 cups/day	1.82	0.49	6.83	0.374
Family history of back pain	2.05	0.62	6.79	0.239
Constant	15.090			0.049

## Discussion

The current study aimed to assess the prevalence and risk factors associated with lower back pain among medical students. Our findings indicated that low back pain was highly prevalent among the medical student population, with 94% of participants reporting experiencing low back pain, and 90% of those cases were not related to trauma. These results are consistent with previous studies that have shown a high prevalence of low back pain among medical students, which can have significant implications for their academic performance and quality of life [2-5].

One of the key findings of our study was that sitting for prolonged periods was identified as a significant risk factor for low back pain among medical students. This finding is supported by previous research that has shown that prolonged sitting can cause musculoskeletal disorders, including low back pain [8-10]. Medical students typically spend long hours sitting in lectures and studying, which may explain the high prevalence of low back pain in this population. Therefore, interventions aimed at reducing prolonged sitting and promoting regular breaks for physical activity may help to prevent low back pain in medical students.

Another important finding of our study was that lack of exercise was identified as a significant risk factor for low back pain. This is consistent with previous studies that have shown that physical inactivity is associated with an increased risk of musculoskeletal disorders [11,12]. Medical students may be particularly susceptible to physical inactivity due to the demanding nature of their studies, which may limit their opportunities for physical activity. Therefore, interventions aimed at promoting physical activity, such as exercise programs or walking meetings, may be effective in reducing the prevalence of low back pain in this population.

Our study also found that factors such as stress, wrong sleep position, sedentary lifestyle, and anxiety were identified as significant contributors to low back pain [13,14]. These findings highlight the importance of addressing these factors as potential risk factors for low back pain in medical students. Strategies such as stress management techniques, ergonomic interventions, and sleep hygiene education may be effective in reducing the prevalence of low back pain among medical students [15].

Interestingly, our study did not find any significant differences in the prevalence of low back pain based on gender, GPA, or academic year. These findings are inconsistent with some previous studies that have reported a higher prevalence of low back pain among female medical students or those in higher academic years [2-5]. The lack of association with low back pain may indicate that low back pain is not necessarily related to academic performance in this population. However, further research is needed to fully understand the factors contributing to low back pain among medical students.

Despite the valuable findings of this study, there are some limitations that need to be considered when interpreting the results. The study is based on a cross-sectional design, which limits our ability to draw causal inferences. Additionally, the study was conducted in a single medical school, and the self-reported measures may introduce recall and social desirability biases. Furthermore, the study did not assess the influence of psychological factors on low back pain and did not investigate the effectiveness of different interventions or treatments for low back pain among medical students. Finally, the lack of objective measures such as imaging or physical examination may lead to an underestimation of the prevalence and severity of low back pain. These limitations highlight the need for further research to confirm and extend the current findings, as well as to identify effective strategies for preventing and managing low back pain in medical students.

## Conclusions

This study sheds light on the high prevalence of low back pain among medical students and identifies several risk factors, associated conditions, and impacts of the condition. It highlights the importance of addressing the issue through targeted interventions that promote physical activity, reduce prolonged sitting, manage stress, and promote good posture. Medical schools can play a vital role in implementing these interventions to support their students' physical and academic well-being. By doing so, they can help prevent and manage low back pain among medical students, thus improving their quality of life and performance. Further research is needed to evaluate the effectiveness of these interventions and explore additional strategies to address this common health issue.
